# Accuracy of predictive scores of hemorrhagic transformation in patients with acute ischemic stroke

**DOI:** 10.1590/0004-282X-ANP-2021-0091

**Published:** 2022-03-14

**Authors:** João Brainer Clares de Andrade, Jay Preston Mohr, Muhammad Ahmad, Fabricio Oliveira Lima, Levi Coelho Maia Barros, Gisele Sampaio Silva

**Affiliations:** 1 Columbia University, Department of Neurology, New York NY, USA.; 2 Universidade Federal de São Paulo, São Paulo SP, Brazil.; 3 CMH Lahore Medical College and Institute of Dentistry, Lahore, Pakistan.; 4 Hospital Geral de Fortaleza, Fortaleza CE, Brazil.; 5 Universidade de Fortaleza, Fortaleza CE, Brazil.; 6 Universidade Estadual do Ceará, Fortaleza CE, Brazil.; 7 Hospital Israelita Albert Einstein, São Paulo SP, Brazil.

**Keywords:** Ischemic Stroke, Precision Medicine, AVC Isquêmico, Medicina de Precisão

## Abstract

**Background:**

Hemorrhagic transformation (HT) is a complication in ischemic strokes, regardless of use of reperfusion therapy (RT). There are many predictive scores for estimating the risk of HT. However, most of them include patients also treated with RT. Therefore, this may lead to a misinterpretation of the risk of HT in patients who did not undergo RT.

**Objective:**

We aimed to review published predictive scores and analyze their accuracy in our dataset.

**Methods:**

We analyzed the accuracy of seven scales. Our dataset was derived from a cohort of 1,565 consecutive patients from 2015 to 2017 who were admitted to a comprehensive stroke center. All patients were evaluated with follow-up neuroimaging within seven days. Comparison of area under the curve (AUC) was performed on each scale, to analyze differences between patients treated with recombinant tissue plasminogen activator (tPA) and those without this treatment.

**Results:**

Our dataset provided enough data to assess seven scales, among which six were used among patients with and without tPA treatment. HAT (AUC 0.76), HTI (0.73) and SEDAN (0.70) were the most accurate scores for patients not treated with tPA. SPAN-100 (0.55) had the worst accuracy in both groups. Three of these scores had different cutoffs between study groups.

**Conclusions:**

The predictive scores had moderate to fair accuracy for predicting HT in patients treated with tPA. Three scales were more accurate for predicting HT in patients not treated with tPA. Through standardizing these characteristics and including more patients not treated with RT in a large multicenter series, accurate predictive scores may be created.

## RESUMO

**Background:**

Transformação hemorrágica (TH) é uma complicação frequente no acidente vascular cerebral (AVC) isquêmico independente do uso de terapia de reperfusão (TR). Diversos escores preditivos de TH foram elaborados. Entretanto, a maioria desses escores incluíram pacientes submetidos a TR — o que pode levar à má interpretação do risco de TH nos pacientes não submetidos a TR.

**Objetivo:**

Nosso objetivo é revisar escores preditivos já publicados e analisar a sua acurácia em nossa amostra.

**Métodos:**

Analisamos a acurácia de sete escores. Nosso banco foi criado de uma coorte de 1.565 pacientes consecutivos, admitidos entre 2015 e 2017 em um centro avançado de AVC. Os pacientes realizaram neuroimagem de controle em até sete dias. Uma comparação entre áreas abaixo da curva/característica de operação do receptor (AUC) foi realizada, analisando-se as diferenças entre grupos de pacientes tratados ou não com ativador de plasminogênio tecidual recombinante (tPA).

**Resultados:**

Nosso banco de dados proporcionou informação suficiente para avaliar sete escores, dos quais seis foram aplicados em pacientes tratados ou não com tPA. HAT (AUC 0,76), HTI (0,73) e SEDAN (0,70) foram os escores mais acurados em pacientes não tratados com tPA. SPAN-100 (0,55) teve a pior acurácia nos grupos. Três desses escores apresentaram diferentes valores de corte entre os grupos.

**Conclusões:**

Os escores apresentaram de boa a moderada acurácia na predição de TH em pacientes tratados com tPA. Três escores foram mais acurados em pacientes não tratados com tPA. A parametrização dessas características e a inclusão de mais pacientes não tratados com TR em um estudo multicêntrico poderia levar a escores mais acurados.

## INTRODUCTION

Hemorrhagic transformation (HT) may be a devastating complication of acute ischemic stroke^1^. About 40% of ischemic strokes undergo HT, regardless of use of acute reperfusion therapy (RT)^2^. HT is a significant cause of early mortality in patients with acute stroke. The risk is higher among patients undergoing thrombolytic therapy^2^. The leading cause of HT is postulated to be endothelial dysfunction marked by a catastrophic failure of capillary integrity, which leads to extravasation of blood^3^.

There are many predictive scores for estimating the risk of HT after ischemic stroke^4,5^. However, most of these models include patients treated with reperfusion therapies. Many of these predictive models do not discriminate among the specific subsets of the patient population who did not undergo reperfusion therapy. Thus, some differences in clinical characteristics between patients who were treated with recombinant tissue plasminogen activator (tPA) and those without this treatment could lead to different cutoffs regarding significant risk factors.

We aimed to present a review of nine such predictive scores and to validate them on a specific cohort of patients, considering the acute therapy received (tPA versus no-tPA). We also aimed to analyze the accuracy of such scales in predicting the risk of HT and compare their accuracy, considering the acute therapy received.

## METHODS

### Selection of predictive scores

We performed a search in the PubMed database using the keywords Hemorrhagic Transformation and Score. We found 2,904 papers. Three authors (JBCA, FOL, GSS) selected the papers based on the main objective of our work. We evaluated ten predictive scores of HT that were published up to August 2019, regardless of reperfusion therapies. Scores that included only patients treated with intra-arterial thrombolysis or mechanical thrombectomy only were not included. In the final analysis, we selected 10 studies.

### Dataset collection

We initially included 2,350 consecutive patients from February 2015 to October 2017 who were admitted to a Brazilian comprehensive stroke center. Patients without follow-up neuroimaging within seven days or medical reports not appropriately filled out were excluded (n=785). Eligible patients were treated with IV tPA in accordance with the national protocol^6^. Patients who underwent mechanical thrombectomy were excluded from this analysis.

### Hemorrhagic transformation

Our primary analysis of interest was based on the discriminative ability of these scales for predicting the presence of HT. HT was diagnosed through evidence of blood or hemoglobin products within the new ischemic area^2,7^ on neuroimaging performed up to seven days after admission. Symptomatic and asymptomatic cases were grouped in regression models. All neuroimages were evaluated by radiologists not involved in patient care and who were not aware of the clinical syndrome or functional status. Neuroradiologists and board-certified neurologists with expertise in vascular neurology addressed any discordances about the presence or absence of HT.

### Follow-up neuroimaging

All patients included had at least one follow-up neuroimaging within seven days after their hospital admission. Patients who underwent IV thrombolysis with tPA had follow-up neuroimaging at least within 24 h after admission. Follow-up neuroimaging was performed either as part of the regular etiological workup or due to neurological deterioration. Computed tomography (CT) scans or magnetic resonance imaging (MRI) were acceptable for performing the follow-up.

### Statistical analysis

The accuracy of the predictive scores was attested through receiver operating characteristic analysis (ROC). We produced a ROC curve for each scale. A comparison of area under the curve (AUC) was performed, to analyze differences between groups of patients (treated or not treated with tPA). We reported values for sensitivity and specificity in relation to values corresponding to optimal performance on the ROC curve for any HT. The points of optimal performance were obtained through Youden's test. The AUC for the ROC curves was compared using a chi-square test with an alpha of 0.05, on the results from our separate analyses, through the methodology proposed by DeLong^8^. All probability values were 2-sided, and p<0.05 was considered statistically significant. We performed Hosmer-Lemeshow tests on the performance of each scale against the primary outcome (any hemorrhagic transformation). The analyses were performed using the SPSS software (version 25.5; IBM)

## RESULTS

We had access to individual data for 1,565 consecutive patients with an established diagnosis of acute ischemic stroke who underwent follow-up neuroimaging within seven days after hospital admission. Their median age was 67 years (range: 57 to 76); NIH stroke scale on admission was 13 points (range: 7 to 19) and ASPECTS on admission was 9 points (range: [7 to 10). Males accounted for 60.5% of our sample. The rate of any HT was 23.1% (n=361). In our dataset, 35.1% (n=577) were treated with tPA. Our in-hospital mortality rate was 8.1% (n=130).

Our dataset provided enough data to assess seven scales, among which six were used on patients either treated or not treated with tPA.

Ten published predictive scales describe 21 risk factors of HT which can be grouped into eight sets: epidemiological (age, ethnics and gender); clinical classification and severity (NIH stroke scale/Canadian neurological scale and lacunar syndrome); laboratory findings (glucose on admission, INR and platelet count); neuroimaging findings (early hypodensity, ASPECTS, volume of the ischemic area and hyperdense MCA sign); vital signs (systolic blood pressure and weight); medical history (diabetes mellitus, arterial blood pressure, heart failure, renal impairment, cancer, antithrombotic medicines and baseline disability), atrial fibrillation and time between symptom onset and treatment. The most cited risk factors of HT were neurological severity, age, glucose on admission and neuroimaging findings (early hypodensity or large injured area). These scores are described in Table 1.

**Table 1:** Published predictive scores for hemorrhagic transformation.

Scales	Variables	Type of HT	Criteria	Neuroimaging technique	Time elapsed until follow-up neuroimaging
HTI^4^	ASPECTS, Atrial Fibrillation on ECG, NIHSS, INR (1.26–1.82 and ≤1.25) and hyperdense middle cerebral artery sign	Any	ECASS I	CT only	7–14 days
HAT^9^	Diabetes mellitus or blood glucose on admission (>200 mg/dL), NIHSS (15–19 or >19) and presence of early visible hypodensity on initial head CT scan (regarding involvement of 1/3 of MCA area)	SHT	NINDS	CT or MRI	24–48 h
SEDAN^10^	Age (>75 years), NIHSS (≥10), early infarct signs on initial CT scan, hyperdense middle cerebral artery sign and blood glucose on admission (145-216 and >216 mg/dL)	SHT	ECASS II	CT or MRI	24 h
iSCORE^11^	Age, atrial fibrillation on ECG, chronic heart failure, NIHSS/Canadian neurological scale, preadmission disability, renal dialysis or cancer, male sex, clinical stroke subtype (lacunar or non-lacunar) and blood glucose on admission (≥135 mg/dL)	Any	ECASS II and NINDS	NA	NA
SPAN-100^30^	Age and NIHSS on admission	Any	NINDS	CT only	36 h
HeRS^31^	Age, infarct volume and glomerular filtration rate	Any	ECASS II	MRI only	Seven days
SITS-SICH^32^	Age (≥72 years), antiplatelet (aspirin alone or aspirin + clopidogrel), history of arterial hypertension, NIHSS (7–12 and ≥13), stroke onset to treatment time (≥180 min), SBP (≥146 mmHg), weight (≥95 kg) and blood glucose on admission (≥180 mg/dL)	SHT	SITS-MOST	CT or MRI	22–36 h
MSS^33^	Age (≥60 years), NIHSS (≥10), blood glucose (>150 mg/dl) and platelet count less than 150,000/mm^3^	Any	NINDS	CT only	Up to 36 h
GRASPS^34^	Age, ethnicity (Asian or non-Asian), male sex, NIHSS, SBP and blood glucose on admission	SHT	NINDS	CT or MRI	Up to 36 h
THRIVE^35^	Age (≤59, 60–79, ≥80), NIHSS on admission (≤10, 11–20, ≥21) and comorbidities (arterial hypertension, diabetes mellitus or atrial fibrillation)	SHT or intraparenchymal hematoma type 2	NINDS and SITS	CT only	72 h

GRASPS: get with the guidelines-stroke SICH risk; SBP: systolic blood pressure; HAT: hemorrhage after thrombolysis; CT: computed tomography; MRI: magnetic resonance imaging; MCA: middle cerebral artery; ECG: electrocardiogram; HTI: hemorrhagic transformation index score; iScore: ischemic stroke predictive risk score; MSS: multicenter rt-PA stroke survey group score; SEDAN: blood sugar [glucose] on admission, early infarct signs and [hyper] dense cerebral artery sign on admission computed tomography [CT] head scan, age, and NIHSS; SITS-SICH: safe implementation of treatments in stroke (SITS) symptomatic intracerebral hemorrhage risk score; SPAN-100: stroke prognostication using age and NIH stroke scale; HeRS: hemorrhage risk stratification score; THRIVE: totaled health risks in vascular events; ASPECTS: Alberta stroke program early CT score; SHT: symptomatic hemorrhagic transformation; ECASS: European cooperative acute stroke study; NINDS: national institute of neurological disorders and stroke.

These scores were used on a mean number of 591±191 patients; and the time that elapsed until follow-up neuroimaging was performed was 192±144 hours, from the time of admission. The overall prevalence of HT ranged from 9.9 to 23.8%, and the prevalence of symptomatic HT ranged from 6 to 12.5% (Table 2).

**Table 2:** Overview of predictive scales.

Scale	AUC – original derivation cohort	AUC – original validation cohort	Points (range)	Cutoff point	Patients not treated with RT included?	Overall prevalence of HT, %	Prevalence of SHT, %	Sample size
HTI^4^	0.85 (0.82–0.89)	0.83 (0.78–0.88)	0–8	2	Yes	23.8	12.5	783
SPAN-100^30^	0.72 (0.63–0.76)	0.59 (0.53–0.65)	0–1	100	Yes	9.9	NA	624
SEDAN^10^	0.77 (0.71–0.83)	0.82 (0.76–0.87)*	0–5	**	No	NA	7	1,813£
GRASPS^34^	0.70 (0.68–0.73)	0.68 (0.66–0.74)	0–101	45–101**	No	NA	4.8	10,242
HAT^9^	0.74 (0.65– 0.79)	0.82 (0.77–0.88)*	0-5	4	No	20	7	400
MSS^33^	0.68 (0.67–0.72)	0.72 (0.67–0.77)*	0-4	3	No	NA	6	1205
SITS-SICH^32^	0.71***	0.69***	0-12	3	No	NA	7.4¥	31,627£
iSCORE^11^	NA	0.83 (0.79–0.87)*	0–180α	>140	No	12.4	6.9	1,696
HeRS^31^	0.701***	0.81 (0.75–0.86)*	-	**	Yes	22	NA	345
THRIVE^35^	0.63***	NA	0–9	2–3**	Yes	6.0	1.6	6,483£

* Validation performed by other authors^4^;

** There is not a single cutoff;

*** Confidence interval is not available;

£ Including validation cohort;

¥ Per NINDS classification;

α The final score ranges from the patients’ age; AUC: area under the curve; GRASPS: get with the guidelines-stroke SICH risk; HAT: hemorrhage after thrombolysis; HTI: hemorrhagic transformation index score; iScore: ischemic stroke predictive risk score; MSS: multicenter rt-PA stroke survey group score; SEDAN: blood sugar [glucose] on admission, early infarct signs and [hyper] dense cerebral artery sign on admission computed tomography [CT] head scan, age, and NIHSS; SITS-SICH: safe implementation of treatments in stroke (SITS) symptomatic intracerebral hemorrhage risk score; SPAN-100: stroke prognostication using age and NIH stroke scale; HeRS: hemorrhage risk stratification score; THRIVE: totaled health risks in vascular events; HT: hemorrhagic transformation; SHT: symptomatic hemorrhagic transformation; NA: not available.

Regarding our primary outcome, HAT^9^, SEDAN^10^ and HTI^4^ were the most accurate predictive scores (Table 3), while SPAN-100^11^ had the smallest AUC. Through formal significance testing, we found that only one scale showed a difference in accuracy between patients treated and not treated with tPA: the HAT^9^ score was more accurate among patients not treated with tPA. None of the other scores differed in accuracy between the two treatment groups.

**Table 3:** Validation in our local dataset (n=1,565).

Scale	Number of patients not treated with RT	AUC	Cutoff point	Sensitivity/specificity, %	Number of patients treated with RT	AUC	Cutoff point	Sensitivity/specificity, %	p-value*
HAT^9^,£	988	0.76 (0.72–0.80)	2	88.4/54.2	577	0.68 (0.62–0.73)	3	51.2/80.1	0.01
HTI^4^	413	0.73 (0.68–0.79)	5	83/54.8	NA	NA	NA	NA	-
SEDAN^10^,£	988	0.70 (0.66–0.74)	3	61.6/72.8	577	0.71 (0.67–0.76)	3	57.3/75.1	0.81
THRIVE^35^	988	0.66 (0.62–0.70)	4	75.8/54.4	577	0.70 (0.66–0.75)	5	53.8/74.6	0.20
GRASPS^34^,£	943	0.64 (0.59–0.68)	70	68/55.3	568	0.68 (0.63–0.73)	74	61.5/68.7	0.22
MSS^33^,£	584	0.60 (0.54–0.65)	2	70.7/44	134	0.60 (0.50–0.71)	2	80.6/58.2	0.95
SPAN-100^30^	988	0.55 (0.51–0.60)	73	67.4/41.1	577	0.63 (0.58–0.68)	73	78.9/35.7	0.80

* Comparing the values for the area under the curve between the groups of patients;

£ Patients not treated with tPA were not included in the original cohort; AUC: area under the curve; GRASPS: get with the guidelines-stroke SICH risk; HAT: hemorrhage after thrombolysis; HTI: hemorrhagic transformation index score; iScore: ischemic stroke predictive risk score; MSS: multicenter rt-PA stroke survey group score; SEDAN: blood sugar [glucose] on admission, early infarct signs and [hyper] dense cerebral artery sign on admission computed tomography [CT] head scan, age, and NIHSS; SITS-SICH: safe implementation of treatments in stroke (SITS) symptomatic intracerebral hemorrhage risk score; SPAN-100: stroke prognostication using age and NIH stroke scale; HeRS: hemorrhage risk stratification score; THRIVE: totaled health risks in vascular events; RT: reperfusion therapy; NA: not available.

Comparative analysis of the scores in the two groups (treated or not treated with tPA) showed that three of them (HAT, HTI and SEDAN) had similar predictive values among patients not treated with RT. Among patients treated with RT, there was no difference in accuracy between the scales (Table 4).

**Table 4:** Comparison of area under the curve between predictive scales of hemorrhagic transformation.

Comparison	Patients not treated with RT	Patients treated with RT
	p-value	p-value
HAT×SEDAN	0.25	0.65
HAT×GRASPS	<0.001	0.67
HAT×SPAN-100	<0.001	0.10
HAT×THRIVE	0.03	0.67
HAT×MSS	<0.001	0.07
HAT×HTI	0.54	-
HTI×SEDAN	0.68	-
HTI×GRASPS	0.001	-
HTI×SPAN-100	<0.001	-
HTI×MSS	0.01	-
HTI×THRIVE	0.09	-
SEDAN×GRASPS	0.004	0.96
SEDAN×SPAN-100	<0.001	0.18
SEDAN×THRIVE	0.09	0.80
SEDAN×MSS	0.002	0.13
GRASPS×SPAN-100	<0.001	0.09
GRASPS×THRIVE	0.27	0.86
GRASPS×MSS	0.94	0.14
SPAN-100×THRIVE	<0.001	0.07
SPAN-100×MSS	<0.001	0.77
THRIVE×MSS	0.06	0.10

AUC: area under the curve; GRASPS: get with the guidelines-stroke SICH risk; HAT: hemorrhage after thrombolysis; HTI: hemorrhagic transformation index score; iScore: ischemic stroke predictive risk score; MSS: multicenter rt-PA stroke survey group score; SEDAN: blood sugar [glucose] on admission, early infarct signs and [hyper] dense cerebral artery sign on admission computed tomography [CT] head scan, age, and NIHSS; SITS-SICH: safe implementation of treatments in stroke (SITS) symptomatic intracerebral hemorrhage risk score; SPAN-100: stroke prognostication using age and NIH stroke scale; HeRS: hemorrhage risk stratification score; THRIVE: totaled health risks in vascular events; RT: reperfusion therapy.

## DISCUSSION

Validation in multicenter samples and comparison of the accuracy of predictive scores of HT are valid resources for choosing the most accurate predictive or diagnostic tool^5^. Studies comparing these scores in different populations of tPA-treated patients have shown similar predictive values. However, there is still no data about these validations among patients not treated with tPA.

Among our patients not treated with RT, three scales showed the best accuracy (HAT, HTI and SEDAN)^4,9,10^. Each of these scales included variables from three categories of predictors: clinical, neuroimaging and laboratory or electrocardiogram. All of these variables can be obtained upon patient admission. All the predictors included in these scores were also reported as significant risk factors of HT in a recent metanalysis, which found 18 variables statistically associated with HT among patients treated with tPA, and 12 of these were listed as risk factors of HT in 10 predictive scores (Table 1).

Besides the quantitative data, reproducibility of the variables included in the predictive models is also essential. Thus, a qualitative analysis may also be helpful in that process. Differences in the clinical and radiological criteria of HT have led to attempts to create scores that integrate multiple factors, in order to better predict the risk of HT.

In our sample, the HAT score was the most accurate score for predicting HT among patients who were not treated with RT, in comparison with those treated with tPA, even though it was developed and tested exclusively for patients who were given IV-tPA. We suppose that the presence and extent of a well-defined ischemic area was a significant factor in distinguishing its accuracy in relation to the two groups. Patients not eligible for RT usually came to the hospital more than 4.5 h after the onset of symptoms, which may have led to a well-defined ischemic area on CT scans. Thus, patients with visible hypodensity on CT scans and large lesions (>1/3 of the area of the middle cerebral artery) had higher scores and, therefore, a higher chance of HT. Moreover, all other variables included in the HAT score were previously described, such as risk factors of HT among patients not treated with tPA^12–17^. The two other most accurate scales among patients not treated with tPA also included previously reported risk factors of HT^12,13,15,17–24^. The inclusion of these risk factors may explain the high accuracy that we found in our sample.

SPAN-100^11^ had an AUC that was smaller than that of the other scores, among our patients not treated with tPA. This scale included just two predictors (age and NIH stroke scale), among which age is taken to be an unclear predictor of HT by some authors^4,25–27^. On the other hand, although SPAN-100^11^ had the same cutoff between the groups, higher sensitivity was found in tPA-treated patients. This finding suggests that there is a need to pay attention to older patients with high NIHSS scores who are eligible for IV tPA.

Our results emphasize the value of external validation of prognostic scales, given that most of our results had accuracy values that were lower than those reported in the original derivation articles. We can infer that predictive scores are most accurate and reliable when patients from different centers and countries are included^28^. Also, we found that the scales had differences in discriminative properties among different samples or groups (i.e. patients treated or not treated with tPA).

Our study had several limitations. First, there was no blinded examiner to attest to the presence of HT, which was confirmed by a radiologist or a neurologist who was board-certified in Brazil. Second, we classified all patients as having any HT or no HT; we did not adopt the clinical classification of HT. Third, we only considered the HT criteria adopted in the ECASS II study^29^. Lastly, our dataset did not contain enough data to provide analysis on three published scales^30–32^.

In conclusion, some of the currently available predictive scores of HT in the literature have moderate to fair accuracy for predicting HT, both among patients treated with tPA and among those without this treatment. This middling level of accuracy may be explained by some disparities in the clinical and radiological classification of HT and the time taken and technique used for neuroimaging follow-up. Considering HT in general, all the predictive scores evaluated had the same accuracy among patients treated with RT; however, among patients not treated with RT, three scales were most accurate for predicting HT.

Through standardizing these characteristics and including patients not treated with RT in a large multicenter series, more accurate predictive scores may be created.
